# Mechanism of an integrated PBL-KAP-AI spiral health education model for breast cancer patients

**DOI:** 10.3389/fpubh.2025.1687956

**Published:** 2025-12-01

**Authors:** Heyu Li, Yang Lu, Yang Tian, Li Zhou, Danna Wang

**Affiliations:** 1 Faculty of Health, Zhejiang Zhoushan Tourism and Health College, Zhoushan, China; 2 Department of Obstetrics and Gynecology, The Second Affiliated Hospital of Harbin Medical University, Harbin, China; 3 Collage of Humanities and Law, Nanchang Institute of Technology, Nanchang, China; 4 Nursing Department, Women's Hospital School of Medicine, Zhejiang University, Hangzhou, China

**Keywords:** breast cancer, health education, problem-based learning (PBL), knowledge–attitude–practice (KAP), artificial intelligence

## Abstract

**Background:**

Breast cancer ranks as the most prevalent cancer among women globally, with significant variations in both incidence and mortality rates. Traditional health education improves knowledge and some behaviors but often fails to sustain long-term change due to its static and unidirectional nature. To overcome these limitations, we developed a spiral health education model integrating Problem-Based Learning, the Knowledge–Attitude–Practice framework, and Artificial Intelligence (PBL–KAP–AI).

**Objective:**

This study aims to assess how the PBL–KAP–AI model influences health behaviors, health beliefs, and quality of life among breast cancer patients, while also exploring the potential mediating and moderating mechanisms involved.

**Methods:**

A randomized trial with implementation limitations was conducted from January to March 2024, involving 488 breast cancer patients. Participants were randomly assigned to either the intervention or control group, with 244 participants in each group. Assessments were carried out pre- and post-intervention (3 months), evaluating health behaviors (HPLP-II), health beliefs, and quality of life (SF-36). Data were analyzed using *t*-tests, repeated measures ANOVA (RM-ANOVA), and Bootstrap-based mediation and moderation models. Strict blinding and allocation concealment were maintained throughout the study, with intervention quality ensured through random audits, AI record reviews, and participant interviews. Intent-to-treat (ITT) analysis was used for handling dropouts, ensuring the integrity of the data and the reliability of the results.

**Results:**

The intervention group achieved significantly greater improvements in health behaviors (123.01 ± 9.90 vs. 98.12 ± 7.96), health beliefs (128.69 ± 15.89 vs. 107.65 ± 16.24), and quality of life (80.58 ± 7.28 vs. 71.37 ± 10.21; all *p* < 0.001). Mediation analysis showed satisfaction influenced quality of life through health behaviors, while moderated mediation revealed behavior levels shaped the pathway to health beliefs.

**Conclusion:**

The PBL–KAP–AI model effectively enhances behaviors, beliefs, and quality of life, offering an interactive, adaptive, and sustainable paradigm for digital health education.

## 1 Introduction

Breast cancer represents the leading cancer diagnosis among women worldwide and ranks second among all cancers. In 2022, it accounted for an estimated 2.3 million new cases and 670,000 deaths globally. Notably, both incidence and mortality show marked regional differences (1, 2). To address this burden, the World Health Organization (WHO) introduced the Global Breast Cancer Initiative (GBCI) in 2021, with the goal of reducing global mortality by 2–4% each year through strategies such as early detection, accurate diagnosis, and standardized treatment (1, 3). Despite advances in therapeutic strategies, outcomes still rely heavily on patients’ sustained adherence to treatment and lifestyle management (i.e., completion of therapy and long-term self-care) (1). This highlights a critical challenge for public health and nursing practice: how to consistently strengthen the “knowledge–belief–behavior” continuum and ultimately improve quality of life.

Prior research has consistently shown that health education in oncology care improves patient understanding and specific behavioral outcomes. However, conventional approaches—characterized by static, unidirectional information delivery—struggle to maintain long-term behavioral change, while insufficient personalization and limited real-time feedback further restrict scalability (4). In the fields of nursing and medical education, research has demonstrated that Problem-Based Learning (PBL) significantly enhances learning motivation, critical thinking, and self-directed learning abilities. Specifically, systematic reviews and meta-analyses have shown that PBL not only boosts students’ motivation to learn but also leads to notable improvements in critical thinking and self-directed learning. Given these advantages, PBL provides a methodological foundation for patient-centered, participatory education (5, 6). Similarly, the Knowledge–Attitude–Practice (KAP) framework has been applied in perioperative and follow-up management of breast cancer. For example, a KAP-based lymphedema prevention program demonstrated improvements in patient behaviors and clinical outcomes, supporting the feasibility of structured, pathway-based approaches (7). Nevertheless, how to integrate participatory learning with behavioral pathways into a closed-loop system that enables individualized and dynamic feedback remains a major limitation of existing models (4).

In the field of oncology education, numerous systematic reviews and meta-analyses have provided important guidance for educational interventions. However, beyond these aggregate analyses, randomized controlled trials (RCTs) play a crucial role as the gold standard for directly validating the effectiveness of educational interventions. Recent RCTs have demonstrated that personalized educational interventions significantly improve health behaviors and quality of life in breast cancer patients (8–10). These studies provide strong evidence for the effectiveness of oncology education interventions and offer theoretical support and design references for the current study.

Recent progress in digital health has created novel opportunities for integrating technology into patient care. Just-in-Time Adaptive Interventions (JITAI) focus on providing personalized support at the optimal moment, leveraging sensor and contextual data to tailor interventions (11). Similarly, reinforcement learning approaches have been successfully applied in behavioral medicine, demonstrating that dynamically adjusting message content through a “behavioral response → message selection” cycle can enhance medication adherence and promote healthier behaviors (12). These approaches are grounded in well-established behavior change theories: Fogg’s Behavior Model suggests that behavior emerges when motivation, ability, and triggers coincide (13), while the COM-B framework elaborates that Behavior is shaped by Capability, Opportunity, and Motivation, offering a strong theoretical basis for integrating technology-driven feedback into the knowledge–attitude–behavior process (14).

Although AI-driven health intervention methods have made significant progress in behavioral health management, and similar intervention models have been applied in several studies, the innovation of this research lies in its unique integration approach. By combining the Problem-Based Learning (PBL) model, the Knowledge-Attitude-Practice (KAP) framework, and AI-driven dynamic feedback mechanisms, we not only offer a new theoretical perspective but also delve into the actual needs of breast cancer patients. This interdisciplinary integration enhances the intervention’s effectiveness while specifically addressing ethical issues related to technology, such as privacy protection and algorithm fairness, thus filling a gap in the existing literature. Therefore, this study not only proposes an innovative approach to behavioral health intervention but also provides valuable insights into the ethical considerations of technological applications.

In summary, the “PBL-KAP-AI Spiral Health Education Model” proposed in this study forms a closed-loop iterative process of “PBL-guided—KAP-deepened—AI-feedback” by integrating participatory learning through PBL, structured pathways via KAP, and dynamic feedback through AI. This approach ensures that educational interventions can meet the individualized needs of patients and achieve continuous optimization. The core advantages of this model are its personalized, real-time, and adaptable health education, which significantly enhances patients’ learning motivation, health beliefs, behaviors, and quality of life.

**Figure 1:**
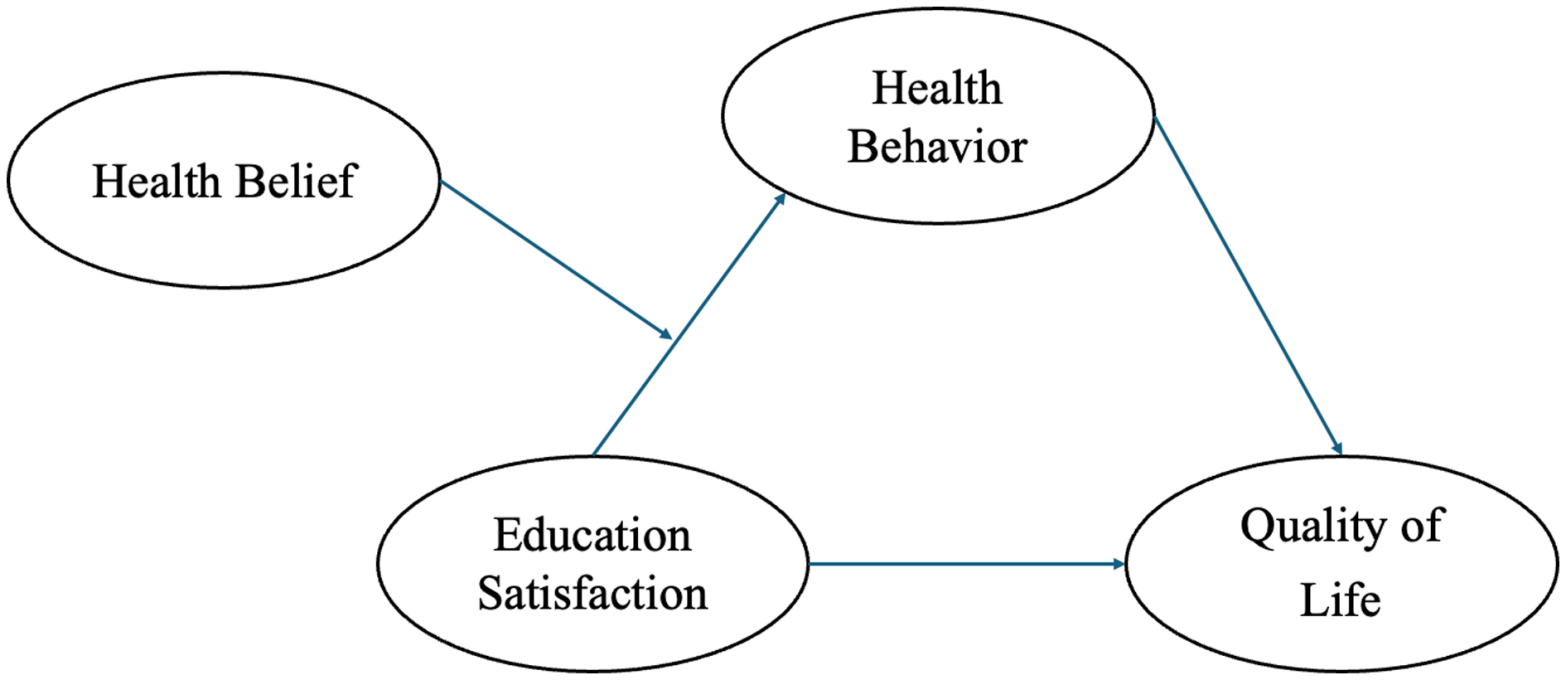
Research model.

Based on this, we construct and validate the “PBL-KAP-AI Spiral Health Education Model,” starting with patient-centered issues (PBL) to stimulate self-directed learning, then translating knowledge into attitudes and practices through the KAP pathway, and finally providing personalized feedback, emotional and status recognition via AI to complete the educational intervention loop. Figure 1 illustrates the hypothetical model of this study.

*H1*: The PBL-KAP-AI model significantly improves the quality of life in breast cancer patients.

*H2*: The PBL-KAP-AI model significantly improves the health behaviors and health beliefs of breast cancer patients, with health behavior mediating the relationship between education satisfaction and quality of life, and health belief moderating the relationship between education satisfaction and health behavior.

## 2 Theoretical background

In this context, the theoretical foundation of this study is derived from three complementary components: Problem-Based Learning (PBL), which enhances patients’ learning motivation and engagement by promoting active exploration; the Knowledge-Attitude-Practice (KAP) model, which provides a progressive pathway from cognition to behavior, aiding in the consolidation of learning outcomes and driving behavioral change; and the AI dynamic regulation mechanism, which serves as a path moderator, enhancing the adaptability and effectiveness of the learning and intervention process through real-time personalized optimization and closed-loop iteration.

### 2.1 PBL: a catalyst for agency and self-directed learning

PBL engages learners through authentic problem scenarios that stimulate motivation and active inquiry, thereby reinforcing critical thinking, autonomous learning, and contextual transfer. Substantial empirical evidence and systematic reviews have demonstrated that PBL significantly enhances higher-order competencies and engagement (5, 6, 15, 16). In patient education, the PBL cycle of “problem–search–integration–reflection” closely mirrors real-world decision-making in disease management, providing a contextualized knowledge base that supports the transition from attitudes to behaviors. By addressing real-world issues faced by patients, PBL stimulates learning motivation, strengthens their self-directed learning abilities, and encourages active decision-making in breast cancer management. PBL emphasizes problem-solving and critical thinking, enabling patients to gain a deeper understanding of their disease and treatment, thereby effectively enhancing their health management behaviors.

### 2.2 KAP model: a staged pathway of “knowing–believing–doing”

The KAP framework emphasizes that knowledge acquisition (K) must be internalized as attitudes or beliefs (A) before being translated into practice (P). Across diverse populations, KAP-based health education has been shown to improve cognition, adherence, and disease control (17–19). A systematic review further confirmed that structured education can significantly enhance disease understanding and self-management among breast cancer patients (20). Nevertheless, conventional KAP pathways often progress in a linear, one-directional manner, with limited attention to time-varying states (e.g., emotions, contexts, accessibility) or stage-specific needs. This frequently results in a “knowing but not doing” gap. This model emphasizes that knowledge acquisition (K) serves as the starting point for behavior change, with knowledge being internalized into attitudes (A), ultimately facilitating the transformation of behavior (P). In breast cancer education, the KAP model assists patients in progressing from understanding the disease and forming accurate beliefs to adopting proactive health behaviors, thereby achieving comprehensive improvements in disease management.

### 2.3 AI as a dynamic regulatory mechanism: a data-driven decision engine of “when, to whom, what, and how much”

Recent frameworks such as Just-in-Time Adaptive Interventions (JITAI) and Micro-Randomized Trials (MRT) have provided theoretical and methodological foundations for real-time, personalized delivery using mobile and wearable data (11, 21, 22). Combined with Dynamic Treatment Regimes (DTR) and reinforcement learning, these approaches enable continuous learning of optimal strategies in real-world settings, supporting adaptive decision-making (12, 23). Randomized and real-world studies have shown that reinforcement learning–based personalization improves adherence to critical health behaviors, validating the feasibility and value of AI as a regulatory pathway (13). Within the broader context of behavior change theories, Fogg’s Behavior Model posits that behavior occurs when motivation, ability, and triggers converge (14); AI can augment this process by optimizing triggers and execution capacity through personalized prompts, emotion recognition, and behavior prediction following PBL-driven motivation. Similarly, the COM-B model highlights the synergy of Capability, Opportunity, and Motivation in shaping behavior, which AI can dynamically modulate across the knowledge, attitude, and practice stages to enhance intervention outcomes (24). Furthermore, Stacey et al. reported in a systematic review that structured integration of behavior change techniques significantly improves cancer survivors’ quality of life, providing additional theoretical support for AI-enabled, multi-stage interventions (25).

In the PBL-KAP-AI Spiral Health Education Model, artificial intelligence (AI) plays a crucial role. The AI platform analyzes patients’ clinical, demographic, and behavioral data to deliver personalized health education tasks and activities, such as micro-courses, case simulations, and group discussions. The content is dynamically adjusted based on the patient’s needs and progress. Additionally, AI integrates multimodal emotion and behavior recognition technologies to monitor patients’ emotional fluctuations in real time and adjusts the educational content accordingly, ensuring the precision and effectiveness of the intervention. Furthermore, by collecting patients’ interaction data, AI optimizes the frequency and pathways of content delivery, enhancing intervention effectiveness. The AI-driven incentive mechanisms also boost patients’ learning motivation, facilitating the transformation of health behaviors and improving self-management capabilities.

This study, based on the PBL-KAP-AI Spiral Health Education Model, proposes a personalized, real-time feedback health education framework by integrating Problem-Based Learning (PBL), the Knowledge-Attitude-Practice (KAP) model, and AI-driven dynamic regulation mechanisms. Unlike traditional linear health education models, the core of the spiral model lies in its nonlinear iterative process. In this process, patients undergo multiple educational cycles, with each cycle encompassing three stages: PBL-guided learning, KAP-based knowledge and belief deepening, and dynamic AI feedback, thereby facilitating self-improvement and continuous optimization.

Throughout the intervention, patients experience several iterations, with the initiation of each iteration determined by AI’s assessment of the patient’s learning progress, health behavior, and emotional state, rather than being solely driven by fixed time points or events (such as behavior cue failure). AI plays a crucial role in this process: it not only dynamically adjusts the content, frequency, and mode of delivery based on the patient’s specific needs but also modifies the difficulty of educational tasks and feedback mechanisms in response to the patient’s reactions, ensuring the intervention is tailored to everyone’s needs. This data-driven real-time optimization process enhances the intelligence and flexibility of the educational model, significantly improving the educational outcomes and driving health behavior changes in patients.

**Figure 2:**
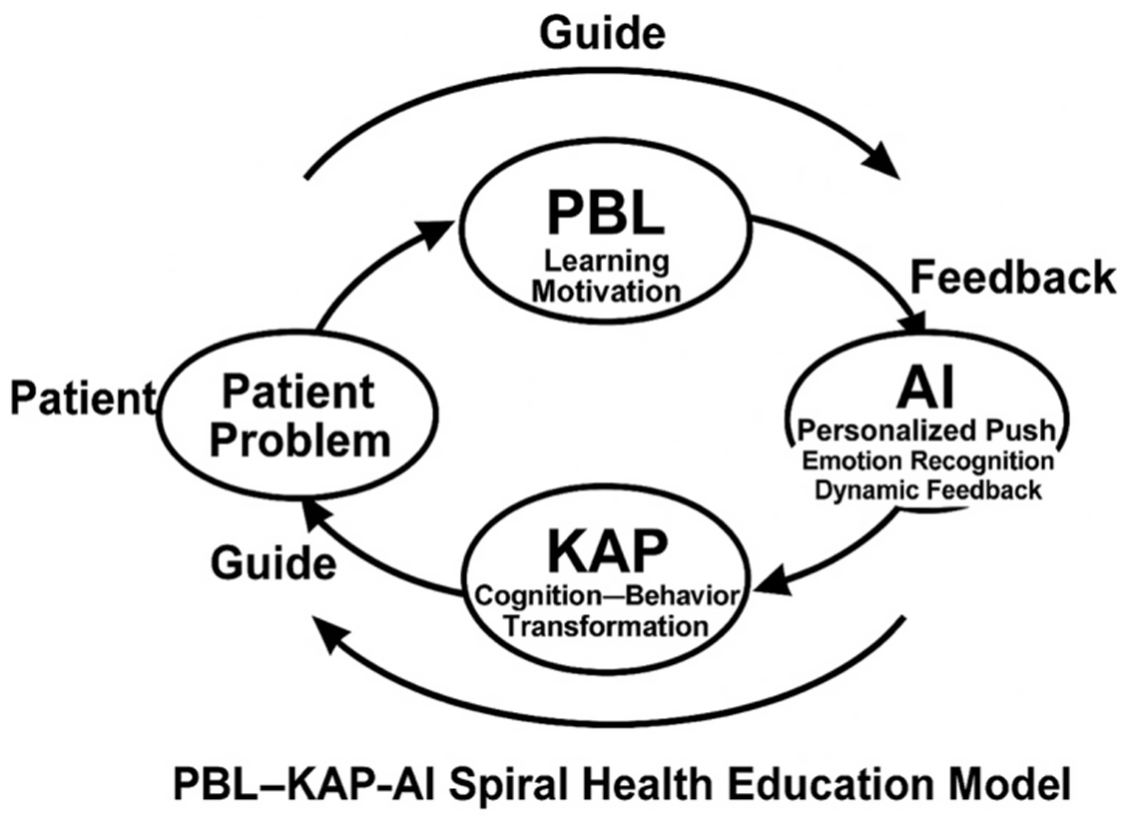
PBL–KAP–AI spiral health education model.

Figure 2 illustrates the nonlinear iterative process of the PBL-KAP-AI model. In contrast to traditional linear educational pathways, our model ensures continuous updates and optimizations of the intervention through its spiral iteration, with each iteration consolidating existing knowledge and beliefs while offering new learning motivation and opportunities for behavioral change. The pathway depicted in the figure clearly demonstrates the cycle from PBL to KAP and AI feedback, highlighting the personalized adjustments and feedback mechanisms at each stage, and emphasizing the dynamic and adaptive nature of the educational process.

## 3 Methods

### 3.1 Study design

This randomized trial with implementation limitations was conducted at a tertiary hospital between January and March 2024 to evaluate the effects of the PBL–KAP–AI spiral health education model on improving health behaviors, health beliefs, and quality of life in breast cancer patients, compared with conventional health education.

### 3.2 Participants

Eligible participants were female breast cancer patients hospitalized at the study site.

Inclusion criteria: (i) age ≥18 years, pathologically or cytologically confirmed breast cancer; (ii) clinically stable, with an expected survival >6 months; (iii) intact communication ability and willingness to complete intervention and assessment.

Exclusion criteria: (i) metastatic cancer or comorbid malignancies; (ii) diagnosed psychiatric disorders or recent major psychological trauma; (iii) GHQ-12 screening score above threshold.

### 3.3 Recruitment and randomization

A total of 488 patients were included in this study. After signing the informed consent form, participants were randomly assigned to either the intervention group or the control group using a computer-generated random number table, with 244 participants in each group. To maintain allocation concealment, sequentially numbered opaque sealed envelopes were used to ensure that the assignment process was unpredictable to the researchers, thereby preventing selection bias. Blinding was applied during the allocation process, ensuring that the researchers were unaware of the group assignments, reducing the risk of selection bias and ensuring the randomness and fairness of the allocation.

Although the allocation method effectively addressed selection bias, potential performance and detection biases may still exist. Performance bias may arise from differences in patients’ perceptions and responses to the intervention, which could affect their behavior and self-reported outcomes. To minimize this bias, strict standardization procedures and training were implemented during data collection. Detection bias could occur if the assessors were aware of patient group assignments, potentially influencing the evaluation of outcomes. While efforts were made to implement blinding, ensuring that outcome assessors and data analysts remained blinded throughout the process, it remains challenging to fully eliminate such bias in practice. To further control these biases, rigorous oversight mechanisms, including random audits, AI-pushed record reviews, and participant interviews, were employed to ensure the objectivity of the data and the reliability of the results.

This study is classified as a “randomized trial with implementation limitations” because, despite the use of computer-generated random allocation and blinding controls, several unavoidable challenges arose during the implementation phase. These challenges include individual variations in patient responses to the intervention (performance bias) and difficulties in fully maintaining blinding during the assessment process (detection bias). Such factors have the potential to impact the integrity and fairness of the study results. Consequently, we have designated this study as a “randomized trial with implementation limitations” to reflect these inherent constraints in the practical application of the trial methodology.

Baseline comparisons revealed no significant statistical differences between the two groups in terms of age, years of education, cultural background, and occupation (*p* > 0.05), indicating that the two groups were highly comparable on these important characteristics. All patients were assessed both before and 3 months after the intervention. For patients who withdrew during the study, the reasons for withdrawal were recorded in detail, and intention-to-treat (ITT) analysis was performed to ensure the comprehensiveness of the data and the reliability of the results.

### 3.4 Interventions

#### 3.4.1 Intervention group: PBL–KAP–AI spiral health education model

**Figure 3:**
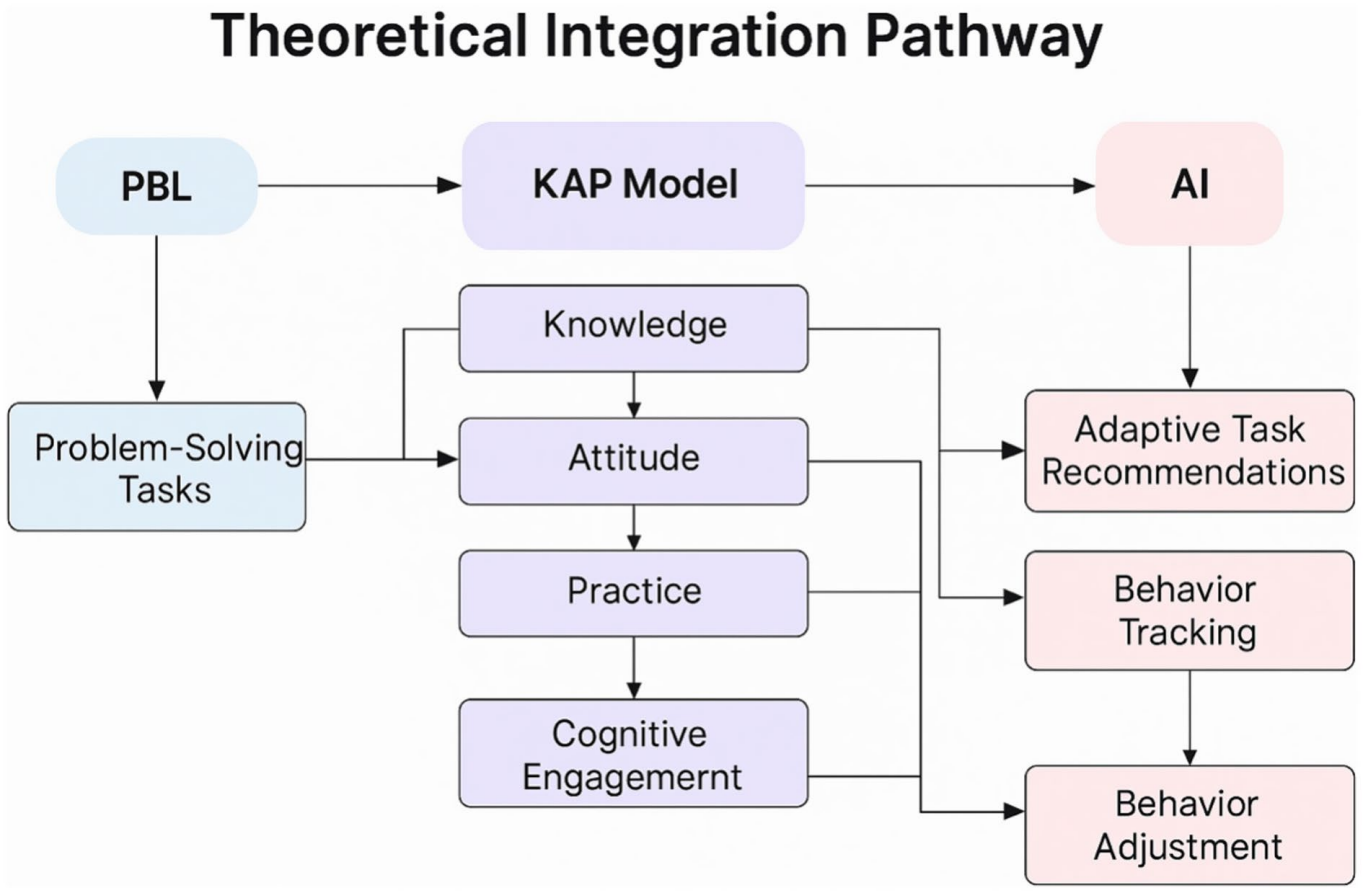
Theoretical integration pathway of the PBL–KAP–AI model.

As illustrated in Figure 3, the model integrates Problem-Based Learning (PBL), the Knowledge–Attitude–Practice (KAP) pathway, and AI-driven adaptive mechanisms to construct a closed loop of “cognitive activation → attitude internalization → behavioral transformation → feedback optimization.”

##### 3.4.1.1 PBL guidance and AI task delivery

The AI platform, informed by patients’ clinical, demographic, and behavioral data, applied collaborative filtering and natural language processing algorithms to provide personalized tasks, including micro-lectures, case simulations, group discussions, and real-time feedback questionnaires. Patients actively participated in PBL discussions under nurse facilitation.

##### 3.4.1.2 KAP-based staged internalization

Through PBL discussions and AI-assisted resources, patients progressively acquired scientific knowledge (K), developed positive health-related attitudes (A), and practiced health-promoting behaviors (P), supported by AI monitoring and motivational mechanisms.

##### 3.4.1.3 AI-enabled dynamic optimization

Leveraging multimodal behavior and emotion recognition, the platform employed dynamic knowledge graphs and reinforcement learning to adapt push frequency, interaction intensity, and content pathways, achieving personalized optimization.

##### 3.4.1.4 AI platform specifications

In this study, the AI platform serves as a core component of the health education intervention, responsible for optimizing the patient education process through personalized task delivery, emotion and state recognition, behavior prediction, and dynamic feedback mechanisms (26). The operation of the AI platform is based on multiple data sources, including initial patient surveys, behavioral data, emotional data, and physiological data. The initial patient survey collects basic information, health status, and learning preferences through questionnaires and interviews, providing the foundation for subsequent personalized interventions. Behavioral data are collected in real-time through mobile applications and wearable devices, covering activity levels, dietary habits, and sleep patterns. Emotional data are assessed using facial expression analysis and speech recognition technology to evaluate the patient’s emotional state. Physiological data are monitored through wearable devices to track health indicators such as heart rate and blood pressure, offering a comprehensive view of the patient’s health.

By integrating these data, the AI platform uses collaborative filtering and natural language processing algorithms to accurately deliver personalized learning tasks (such as micro-courses, case simulations, and group discussions). The platform combines multimodal behavior and emotion recognition technologies to analyze patients’ emotional responses and activity levels, dynamically adjusting the educational content and feedback strategies to ensure alignment with the patient’s real-time state (27). Additionally, the AI system employs reinforcement learning algorithms to conduct online learning and optimization based on patient feedback (such as health behavior execution and emotional responses), dynamically adjusting the frequency, content, and intensity of task delivery. The AI platform also constructs and updates a “knowledge graph” in real time, adjusting the representation of knowledge based on the patient’s health behaviors and emotional state, ensuring the accuracy and personalization of the content being delivered (28).

The platform’s processing algorithms analyze individual patient characteristics and preferences using collaborative filtering and natural language processing technologies, thereby precisely pushing appropriate health education content. The AI system dynamically adjusts delivery strategies based on the patient’s behavioral responses and learning progress, ensuring that the educational content adapts to the patient’s needs and enhancing the personalization and contextualization of the intervention. By continuously collecting data, the AI platform automatically adjusts the delivery strategy, educational content, and interaction intensity to ensure personalized and real-time feedback. Additionally, through a closed-loop feedback mechanism, the platform continuously optimizes educational content and pathways by collecting real-time feedback on patient behaviors and emotional states, ensuring ongoing improvement in intervention effectiveness (29).

In summary, the PBL-KAP-AI Spiral Health Education Model employed in this study integrates Problem-Based Learning (PBL), KAP behavioral theory, and AI-driven dynamic feedback mechanisms. Unlike traditional health education tools, AI does not merely deliver standardized information. Instead, it dynamically adjusts the intervention content and frequency based on real-time analysis of patient behavior data and emotional states, ensuring a personalized and contextualized health education experience. This process enables AI to optimize in real time based on patient responses, achieving truly intelligent and adaptive health management. The technical roadmap of this study is shown in Figure 3.

The intervention cycle in this study spans 3 months, with two interventions per week, each lasting 45 min, totaling 24 sessions. During each intervention, the AI platform will deliver personalized educational content, incorporating Problem-Based Learning (PBL) discussions and AI feedback to assist patients in gradually transforming their health behaviors. The intervention content includes personalized task delivery, health education micro-courses, case simulations, and more, with patients engaging in learning and interaction via mobile applications and the AI platform. Additionally, patients will participate in monthly PBL group discussions, facilitated by trained nursing staff or research assistants who provide guidance.

To ensure consistency and quality of the intervention, all sessions will be conducted by nursing staff or research assistants who have undergone uniform training and passed consistency assessments. The research team has established stringent oversight mechanisms, including random audits, reviews of AI push notifications, and participant interviews, to ensure the standardization of the intervention process and continuous optimization of its effectiveness.

#### 3.4.2 Control group: conventional health education

In this study, the control group patients will receive conventional health education. This educational model includes a standardized health education manual, health lectures, and WeChat public account content, aimed at providing patients with basic disease knowledge and health management information. The control group will not receive personalized content delivery, Problem-Based Learning (PBL) discussions, or AI tracking. The health education manual covers breast cancer-related information, treatment plans, and lifestyle recommendations, while the health lectures and WeChat content are updated periodically but are not personalized based on the specific needs or progress of the patients.

The intervention cycle lasts for 3 months, with two interventions per week, each lasting 45 min, for a total of 24 sessions. Patients will learn through traditional face-to-face lectures and the manual content, without dynamic personalized feedback. The control group patients will not participate in PBL group discussions or receive interaction and support from the AI platform.

To ensure the standardization and quality of the intervention process, all conventional education sessions will be conducted by nursing staff or research assistants who have undergone uniform training. The research team has established oversight mechanisms, including random audits and participant interviews, to ensure consistency in the intervention content and quality control.

### 3.5 Measurement tools

#### 3.5.1 Health education satisfaction

Satisfaction with the health education intervention was measured using a self-developed questionnaire consisting of 8 items (e.g., “Match between intervention content and patient needs,” “Satisfaction with AI-pushed tasks”). Responses were rated on a 5-point scale (1 = Very dissatisfied, 5 = Very satisfied), with total scores ranging from 8 to 40. Content validity was assessed by 5 experts (2 oncology nursing experts, 2 health education experts, and 1 AI technology expert), yielding a content validity index (CVI) of 0.92. Reliability was tested through a pilot study (*n* = 50), with a Cronbach’s *α* coefficient of 0.86 (30, 31).

#### 3.5.2 Health behaviors

Measured by the validated Chinese version of the Health-Promoting Lifestyle Profile II (HPLP-II), consisting of 52 items across six subscales, each rated on a 4-point scale (32, 33). To ensure the validity of health behavior measurement, the HPLP-II scale used in this study has undergone multiple validations and demonstrates high internal consistency, with a Cronbach’s *α* value above 0.8. Additionally, standardized assessment procedures were employed, and the evaluators, who underwent multiple rounds of training, were carefully selected to minimize bias.

#### 3.5.3 Health beliefs

Evaluated with the Chinese version of the Health Belief Model scale, which includes dimensions of perceived susceptibility, severity, benefits, barriers, self-efficacy, and cues to action (34). In this study, the reported “Health Belief Score” is the sum of the scores from all subscales. Given the high internal consistency of each subscale (Cronbach’s *α* > 0.80), indicating strong correlations between the dimensions, the subscale scores were combined into a total score for analysis.

#### 3.5.4 Quality of life

Assessed using the Chinese version of the SF-36, covering 8 domains. Scores range from 0 to 100, with higher scores reflecting better quality of life (35).

#### 3.5.5 Psychological health screening

Conducted with the Chinese GHQ-12 at baseline to exclude participants with potential psychological disorders (36, 37).

### 3.6 Data collection and analysis

Data collection occurred at two time points: baseline and immediately post-intervention. The baseline time point involved the collection of initial data on patients’ health behaviors, health beliefs, quality of life, and education satisfaction. The post-intervention time point, which took place in the first week following the intervention, assessed the immediate effects of the intervention on patients’ health behaviors, health beliefs, and quality of life.

To account for potential self-report bias, standardized procedures were implemented during data collection to ensure that all patients understood the assessment content. Additionally, to minimize bias, evaluators underwent multiple rounds of training, and the intervention was supplemented by direct behavioral observation and wearable device data for auxiliary validation.

To further validate the robustness of the results, sensitivity analyses will be conducted to assess the impact of varying assumptions or changes in variables on the study outcomes. By analyzing different data handling methods and hypothetical conditions, we aim to ensure the reliability and applicability of the study conclusions.

Data were collected at baseline and again 3 months after the intervention. Analyses were performed using SPSS 26.0. Tests of normality and homogeneity of variance were conducted prior to group comparisons. Between-group differences were analyzed with independent-sample *t*-tests, while repeated measures ANOVA (RM-ANOVA) was applied for within-group comparisons. A significance threshold of *p* < 0.05 was adopted. Mediation and moderated mediation analyses were carried out using PROCESS v4.0 with 5,000 bootstrap samples, testing the mediating pathway of satisfaction (“satisfaction → health behavior → quality of life”) and the moderating influence of health beliefs.

### 3.7 Ethical considerations

The research protocol received approval from the Zhejiang Zhoushan Tourism and Health College (Approval No. [2023-7]). Prior to participation, all individuals were fully informed of the study objectives and procedures and provided written consent. Participation was entirely voluntary, with assurances of confidentiality, anonymity, and the option to withdraw at any stage. The study was conducted in strict compliance with the ethical standards outlined in the World Medical Association’s Declaration of Helsinki (2013 revision).

In this study, we strictly adhered to data protection and privacy principles to ensure the security of all sensitive data. All behavioral and emotion recognition data were anonymized during collection and storage, with personal identifiers removed and stored on encrypted servers accessible only to authorized researchers. We employed encryption technologies that comply with data protection laws, including the GDPR, and conducted regular security audits to ensure the integrity and security of the data. All participant data were kept strictly confidential, with the study results presented in an anonymized format. Participants were fully informed about the data processing procedures and their right to withdraw from the study at any time through the informed consent form. At the conclusion of the study, all data will be securely destroyed in accordance with applicable regulations to ensure full compliance with privacy protection standards.

## 4 Results

### 4.1 General characteristics

As presented in Table 1, baseline comparisons revealed no statistically significant differences between the intervention and control groups (*p* > 0.05). The groups were well balanced with respect to demographic characteristics (age, marital status, occupation, educational level, and socioeconomic status) as well as disease-related factors, indicating satisfactory baseline equivalence (Table 1).

**Table 1:** General information survey of breast cancer patients.

Characteristics		Intervention group	Control group	X^2^	*p*-value
		*n* = 244 (%)	*n* = 244 (%)		
Age	≤30	52 (21.31)	54 (22.13)	0.21	0.98
	31–50	78 (31.97)	80 (32.79)		
	51–65	58 (23.77)	58 (23.77)		
	>65	56 (22.95)	52 (21.31)		
Employment status	Employed	146 (59.84)	139 (56.97)	0.01	0.93
	Not employed	98 (40.16)	105 (43.03)		
Highest education level	Primary school or below	20 (8.20)	26 (10.66)	2.67	0.62
	Middle school	25 (10.25)	18 (7.38)		
	High school	70 (28.69)	78 (31.97)		
	Associate degree	62 (25.41)	56 (22.95)		
	Bachelor’s degree or above	67 (27.46)	66 (27.05)		
Marital status	Married	183 (75.00)	180 (73.77)	0.04	0.84
	Not married	61 (25.00)	64 (26.23)		
Monthly *per capita* household income (USD)	< 700	88 (36.07)	90 (36.89)	0.01	0.99
	701–1,500	104 (42.62)	04 (36.11)		
	>1,500	52 (21.31)	50 (20.49)		
Cancer staging	Stage 0	69 (28.28)	59 (24.18)	6.34	0.71
	Stage I	37 (15.16)	42 (17.21)		
	Stage II	61 (25.00)	62 (25.41)		
	Stage III	77 (31.56)	81 (33.20)		
	Stage IV	0	0		
Treatment stages	New diagnosis stage	78 (31.97)	80 (32.79)	0.05	0.98
	Active treatment stage	104 (42.62)	102 (41.80)		
	Survivorship stage	62 (25.41)	62 (25.41)		
Received health education	Yes	86 (35.25)	82 (33.61)	0.01	0.76
	No	158 (64.75)	162 (66.39)		

### 4.2 Quality of life

To evaluate the impact of the PBL-KAP-AI spiral health education model on the participants’ quality of life, a comparative analysis was conducted between the intervention group and the control group before and after the intervention.

As shown in Table 2, prior to the intervention, the quality of life scores for the intervention and control groups were 37.34 ± 11.17 and 37.28 ± 10.29, respectively, with no statistically significant difference (*t* = 0.07, *p* = 0.95). After the intervention, the intervention group’s score significantly increased to 80.58 ± 7.28, while the control group’s score increased to 71.37 ± 10.21. The difference between the two groups was statistically significant (*t* = 11.47, *p* < 0.001). Within-group comparisons also revealed significant improvements for both the intervention group (*t* = 52.08) and the control group (*t* = 35.93, *p* < 0.001) after the intervention.

**Table 2:** Comparison of quality of life scores between the intervention and control groups before and after the intervention.

Group	Pre-intervention	Post-intervention	*t*-value	*p*-value	Cohen’s d	95% Confidence interval
Intervention group (*n* = 244)	37.34 ± 11.17	80.58 ± 7.28	52.08	0.00	4.59	[4.25, 4.92]
Control group (*n* = 244)	37.28 ± 10.29	71.37 ± 10.21	35.93	0.00	3.33	[3.05, 3.60]
*t*-value	0.07	11.47				
*p*-value	0.95	0.00				

**Figure 4:**
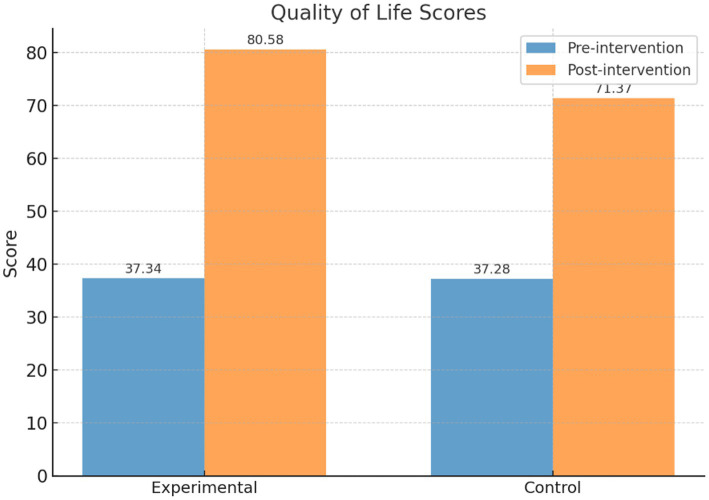
Comparison of quality of life scores between the intervention and control groups.

As shown in Figure 4, the PBL-KAP-AI spiral health education model significantly improved individual quality of life, with a more pronounced effect observed in the intervention group. Therefore, Hypothesis 1 is supported.

### 4.3 Health behaviors and health beliefs

#### 4.3.1 Health behaviors

To evaluate the effect of the PBL-KAP-AI spiral health education model intervention, this study compared the changes in health behavior scores before and after the intervention between the intervention and control groups.

**Figure 5:**
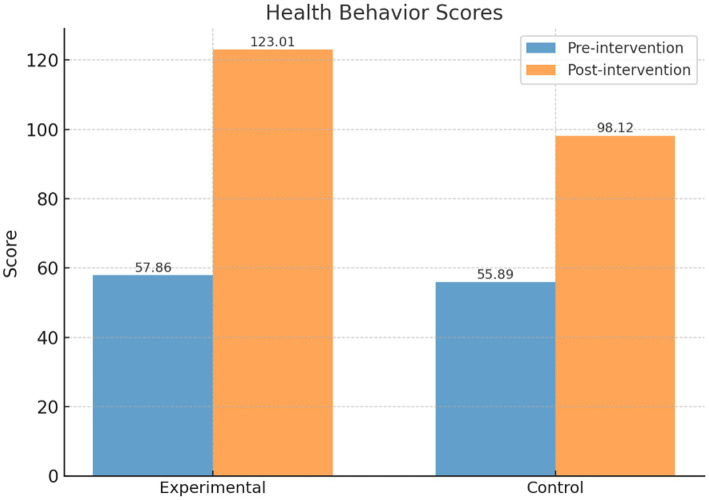
Comparison of health behavior scores between the intervention and control groups.

As shown in Table 3 and Figure 5, prior to the intervention, the health behavior score of the intervention group (*n* = 244) was 57.86 ± 11.72, which significantly increased to 123.01 ± 9.90 post-intervention, with a statistically significant difference (*t* = 64.60, *p* < 0.001). The control group (*n* = 244) had a pre-intervention score of 55.89 ± 10.13, which increased to 98.12 ± 7.96 post-intervention, with a significant difference as well (*t* = 52.43, *p* < 0.001).

**Table 3:** Comparison of health behavior scores between the intervention and control groups before and after the intervention.

Group	Pre-intervention	Post-intervention	*t*-value	*p*-value	Cohen’s d	95% Confidence interval
Intervention group (*n* = 244)	57.86 ± 11.72	123.01 ± 9.90	64.60	0.00	6.01	[5.59, 6.42]
Control group (*n* = 244)	55.89 ± 10.13	98.12 ± 7.96	52.43	0.00	4.64	[4.29, 4.98]
*t*-value	1.98	30.61				
*p*-value	0.05	0.00				

Further comparison of post-intervention differences between the two groups showed that the intervention group scored significantly higher than the control group (*t* = 30.61, *p* < 0.001), indicating that the PBL-KAP-AI spiral health education model had a significant positive effect on improving health behaviors.

In breast cancer patients’ health education interventions, there is often a significant gap between patients’ knowledge and actual behaviors. Therefore, the significant improvement in behavioral scores observed in this study may be attributed to the highly targeted and personalized nature of the intervention, which successfully promoted rapid changes in patients’ health behaviors.

#### 4.3.2 Health beliefs

To investigate the effect of the PBL-KAP-AI spiral health education model intervention on participants’ health beliefs, this study compared the changes in health belief scores before and after the intervention between the intervention and control groups.

**Figure 6:**
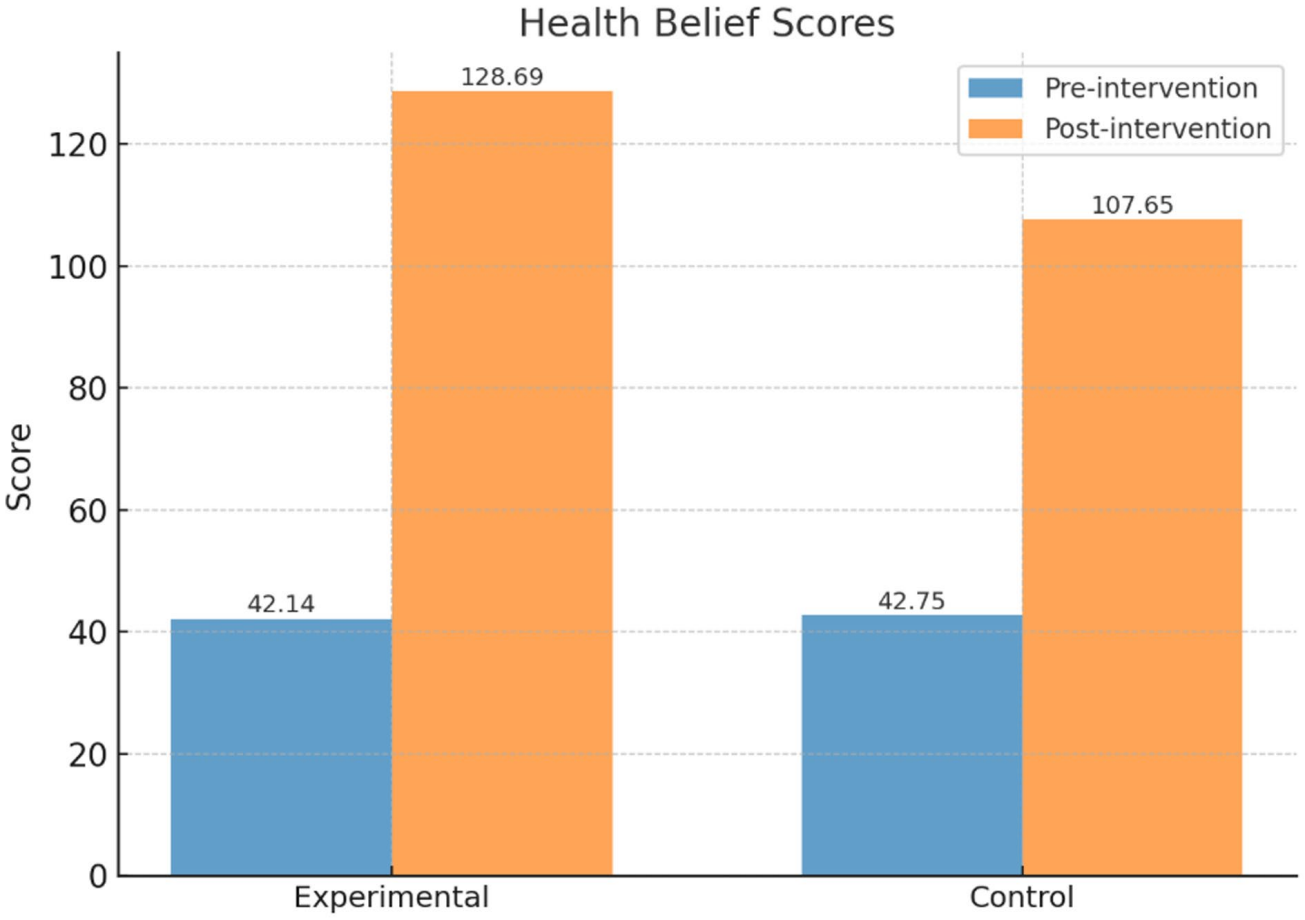
Comparison of health belief scores between the intervention and control groups.

As shown in Table 4 and Figure 6, there was no statistically significant difference between the two groups before the intervention (intervention group: 42.14 ± 7.64, control group: 42.75 ± 4.93, *t* = 1.04, *p* = 0.30). After the intervention, the intervention group’s health belief score significantly increased to 128.69 ± 15.89, while the control group’s score also increased to 107.65 ± 16.24. The difference between the two groups was statistically significant (*t* = 14.47, *p* < 0.001). Within-group comparisons also showed significant improvements in both groups (intervention group: *t* = 88.93; control group: *t* = 60.43, *p* < 0.001).

**Table 4:** Comparison of health belief scores between the intervention and control groups before and after the intervention.

Group	Pre-intervention	Post-intervention	*t*-value	*p*-value	Cohen’s d	95% Confidence interval
Intervention group (*n* = 244)	42.14 ± 7.64	128.69 ± 15.89	88.93	0.00	6.94	[6.47, 7.41]
Control group (*n* = 244)	42.75 ± 4.93	107.65 ± 16.24	60.43	0.00	5.41	[5.03, 5.79]
*t*-value	1.04	14.47				
*p*-value	0.30	0.00				

The results indicate that the PBL-KAP-AI health education model is highly effective in enhancing individuals’ health beliefs, with the intervention group showing significantly greater improvement than the control group.

### 4.4 Mediation effect

This study employed PROCESS macro Model 4 to examine the mediating role of health behavior (M) in the relationship between satisfaction with the PBL–KAP–AI spiral health education model (X) and quality of life (Y), based on a sample of 244 participants.

Results indicated that satisfaction with health education exerted a significant positive predictive effect on health behavior (*β* = 0.29, *t* = 21.01, *p* < 0.001), suggesting that higher satisfaction levels were associated with greater engagement in positive health behaviors. Further analysis revealed that health behavior significantly predicted quality of life (β = 0.51, *t* = 15.39, *p* < 0.001).

Even after controlling for health behavior, the direct effect of satisfaction on quality of life remained significant (β = 0.07, *t* = 5.42, *p* < 0.001), indicating that health behavior served as a partial mediator in this pathway.

As shown in Table 5, the mediation analysis further demonstrated that the indirect effect of satisfaction on quality of life through health behavior was 0.15, with a Bootstrap 95% confidence interval of [0.13, 0.18], excluding zero, thereby confirming the significance of the mediation effect. The completely standardized indirect effect was 0.56, providing additional evidence for the crucial mediating role of health behavior.

**Table 5:** Mediation analysis of health behavior in the relationship between satisfaction with the PBL–KAP–AI spiral health education model and quality of life.

Effect type	Effect	SE	*t*	*p*	95% CI (LL)	95% CI (UL)
Total effect	0.22	0.01	21.27	0.00	0.20	0.24
Direct effect	0.07	0.01	5.42	0.00	0.04	0.09
Indirect effect	0.15	0.01	–	–	0.13	0.18

In summary, the health education satisfaction demonstrated a significant mediating effect on quality of life through health behaviors. Both path a (satisfaction → health behavior) and path b (health behavior → quality of life) exhibited strong positive effects, while path c’ (direct effect of health education satisfaction → quality of life) also showed a significant positive effect. Health behavior played a crucial mediating role in this process, further supporting the mechanism through which increased education satisfaction improves quality of life.

### 4.5 Moderated mediation effect

To examine whether the effect of satisfaction with the PBL–KAP–AI spiral health education model (X) on health beliefs (Y) was mediated by quality of life (M), and whether health behavior (W) moderated this mediating pathway, we conducted a moderated mediation analysis using PROCESS macro Model 7 with a sample of 244 participants.

Regression analyses revealed that health education satisfaction exerted a significant main effect on quality of life (B = −0.96, *p* < 0.001), and health behavior also showed a significant main effect (B = −0.33, *p* < 0.001). Moreover, the interaction term between satisfaction and health behavior significantly predicted quality of life (B = 0.01, *p* < 0.001), indicating that health behavior played a significant moderating role in the relationship between satisfaction and quality of life (Table 6).

**Table 6:** Regression coefficients for the effects of PBL–KAP–AI health education satisfaction, health behavior, and their interaction on quality of life (model 7).

Path	B	SE	*t*	*p*
Constant	150.20	3.73	40.29	0.00
Participation (X)	−0.96	0.06	−14.96	0.00
Collaboration (W)	−0.33	0.03	−11.31	0.00
X × W interaction	0.01	0.00	19.58	0.00

Further conditional indirect effect analyses demonstrated that at low (M − 1 SD), moderate (M), and high (M + 1 SD) levels of health behavior, the indirect effects of satisfaction on health beliefs via quality of life were 0.07, 0.14, and 0.22, respectively. All bootstrap confidence intervals excluded zero, confirming that the mediation effects were significant across different levels of the moderator. The index of moderated mediation was 0.001, with a 95% CI of [0.00, 0.01], further supporting the presence of a significant moderating effect of health behavior on the indirect pathway linking health education satisfaction to health beliefs through quality of life (Table 7).

**Table 7:** Moderated mediation model of health education satisfaction, quality of life, health beliefs, and health behavior.

Collaboration level (W)	Indirect effect	BootSE	95% CI (LLCI)	95% CI (ULCI)
Low (M − 1 SD)	0.07	0.01	0.05	0.08
Medium (M)	0.14	0.01	0.13	0.16
High (M + 1 SD)	0.22	0.01	0.20	0.25

**Figure 7:**
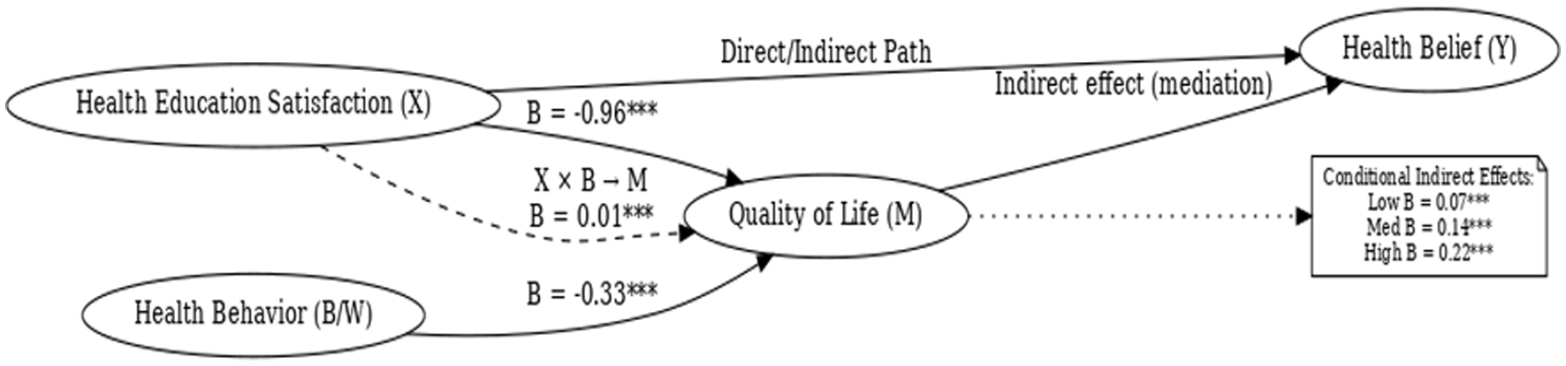
Moderated mediation model of health education satisfaction, quality of life, health belief, and health behavior.

In summary, as shown in Figure 7, this study supports a significant mediation-moderation model. The moderation effect analysis revealed that health behavior level (the moderating variable) plays a significant moderating role in the mediation path between health education satisfaction and health beliefs. Specifically, the indirect effect of health education satisfaction on health beliefs, mediated by quality of life, is moderated by the level of health behavior. That is, at low, medium, and high levels of health behavior, the indirect effect of health education satisfaction on health beliefs is 0.07, 0.14, and 0.22, respectively, indicating that the level of health behavior significantly moderates the strength of this mediation path. In other words, the PBL-KAP-AI spiral health education model satisfaction can enhance individuals’ health beliefs through improvements in their quality of life, and this process is significantly moderated by their level of health behavior.

## 5 Discussion

This study validated the short-term effectiveness of the PBL–KAP–AI spiral health education model among breast cancer patients and further elucidated the unique mechanistic pathways shaped by AI-driven dynamic feedback and health belief modulation. The findings revealed two key mechanisms: first, health behavior partially mediated the relationship between health education satisfaction and quality of life; second, the level of health behavior moderated the indirect effect of satisfaction on health belief through quality of life, such that higher behavioral engagement amplified the impact of satisfaction on both belief and quality of life. In other words, satisfaction must be translated into concrete behavioral change, reinforced by AI-supported dynamic feedback, to meaningfully enhance quality of life and strengthen health beliefs.

In this study, the intervention group demonstrated significant improvements in health behaviors, health beliefs, and quality of life, showing marked differences compared to conventional health education. While these improvements are notable, we acknowledge the potential risk of overestimation. To provide a plausible explanation for these effects, several factors must be considered. First, high initial motivation among participants played a crucial role in driving substantial improvements. Prior to the intervention, participants exhibited a strong desire for health education and a proactive attitude, which likely enhanced the effectiveness of the intervention. Second, the personalized, face-to-face format of the intervention, characterized by its interactive nature, may have facilitated a deeper understanding and better application of the learned knowledge, thereby further amplifying the behavioral changes. Additionally, self-report bias should be considered, particularly in the evaluation of health behaviors and quality of life. Participants may have been influenced by social desirability bias, leading them to report more favorable behaviors and outcomes. Therefore, future research should incorporate more objective physiological data or behavioral tracking to validate these self-reported results.

Although this study validated the short-term effects of the PBL-KAP-AI spiral health education model in breast cancer patients, and the dynamic feedback mechanism of AI contributed to the transformation of health beliefs and behaviors, it is important to note that some studies have shown that AI-driven health interventions do not produce significant effects for all patients. For instance, certain studies have found that AI interventions failed to significantly improve health behaviors or quality of life in some patient groups (38, 39). Furthermore, research has indicated that the effectiveness of AI interventions may be influenced by factors such as patients’ digital literacy, individual differences, and technical issues during the intervention process (e.g., incomplete data, delayed feedback). Therefore, future research should further explore the reasons for these ineffective or negative outcomes and seek strategies to improve the effectiveness of AI interventions.

Compared with existing AI-based health education studies, the present research offers several innovations. Most importantly, it elevates AI from a passive information delivery tool to an embedded dynamic moderator within a theoretically grounded pathway, sustaining the iterative spiral of “cognition–attitude–behavior.” This dynamic regulation mechanism, driven by algorithms for content delivery and behavior tracking, establishes a personalized and adaptive feedback loop, ensuring that the educational content and intervention strategies for each patient are adjusted in real-time based on their responses and progress. While this framework provides important directions for the future development of digital health education, the specific ways in which AI achieves these personalized adjustments still require further detailed clarification. In terms of algorithm implementation, although we employed a personalized feedback mechanism, the specific algorithmic details and the assurance of fairness have not been thoroughly explored. Future research should further describe these algorithms in detail, including how they dynamically adjust the content delivery based on real-time patient feedback, and ensure fairness and effectiveness across different patient groups. Second, health belief was conceptualized as a dual-role construct—both as an outcome variable and as a moderating factor influencing the efficiency of behavior adoption and quality of life improvement. Finally, through a quasi-randomized controlled design combined with mediation and moderation analyses, this study systematically delineated the interactive mechanisms linking satisfaction, behavior, belief, and quality of life. Such a design directly addresses the prevalent “information–behavior gap” in digital health interventions (11, 40, 41).

It is worth noting that PBL, as an instructional approach, carries both strengths and limitations. Prior evidence has shown that blended learning can enhance efficiency and optimize learning depth, providing a feasible strategy for expanding PBL in clinical populations (42). However, its implementation requires considerable organizational and facilitation capacity and may be constrained by disparities in educational resources and learner preparedness.

This study also highlights the importance of addressing digital accessibility, privacy protection, and algorithmic fairness during implementation. Existing evidence suggests that a digital divide still exists among older populations in terms of digital access (43), while the widespread use of health data also brings privacy risks (44). Additionally, if algorithms are not properly scrutinized and corrected, they may exacerbate health inequalities (45). Therefore, future efforts should not only focus on the technological advancements themselves but also implement a range of measures to address these concerns.

To bridge the digital divide, in addition to providing digital literacy training, simplifying device interfaces, and offering technical support, it is important to consider establishing service points at the community level and providing mobile device support to help older adults enhance their digital access capabilities. Regarding privacy protection, data security can be ensured through encryption, data anonymization, and tiered authorization management. Moreover, regular data protection audits should be conducted to ensure the security and privacy of the data.

In terms of algorithmic fairness, it is essential to regularly review and adjust algorithms to ensure fairness across different populations. This can be achieved by building diverse training datasets that cover various age groups, genders, regions, and health statuses, thereby reducing algorithmic bias. Furthermore, establishing an independent algorithm review mechanism, including performance evaluation and feedback, will further ensure fairness in practical applications. These measures will effectively address the ethical issues related to technology application, ensuring that the technology can reach a broader population and reduce potential health disparities.

In terms of effectiveness, this study not only reaffirmed the utility of the KAP model for behavioral improvement but also demonstrated how integrating PBL promotes active knowledge construction and belief activation. Coupled with AI-driven content delivery and behavior tracking, the model established a personalized and adaptive feedback loop. Unlike traditional KAP interventions characterized by linear information transfer, this approach aligns more closely with a “learning-driven–belief-reinforced–behavior-adopted” spiral cycle (46, 47). Furthermore, the findings extend existing research on the relationship between behavioral interventions and quality of life (48–51).

Health behaviors acted as a mediator between education satisfaction and quality of life. Education satisfaction indirectly enhanced the quality of life in breast cancer patients by promoting the adoption of health behaviors. This suggests that when patients are satisfied with the health education they receive, they are more likely to engage in positive health behaviors, such as increasing physical activity and improving dietary habits, which significantly improves their quality of life. This finding is consistent with related research in health behavior change models, which emphasize the important role of health behaviors in promoting quality of life (52). However, the unique contribution of this study lies in the discovery that education satisfaction indirectly influences quality of life through health behaviors, a mechanism that has not been widely explored. Future research could further validate this pathway and investigate the impact of different types of interventions on this mechanism.

The role of health belief in this study was evident not only as an outcome but also as a moderator. Significant differences in the indirect effects of satisfaction on belief across levels of behavior demonstrated that behavior functions simultaneously as a product of belief and as a foundation for its reinforcement. This is consistent with the Health Belief Model, which posits that stronger beliefs significantly increase the likelihood of behavior adoption (53).

This study validated the short-term intervention effects of the PBL-KAP-AI spiral health education model in breast cancer patients and revealed the mediating role of education satisfaction in the improvement of quality of life through health behaviors. The study found that health behaviors played a key mediating role between education satisfaction and quality of life, with health behavior levels further moderating the impact of satisfaction on health beliefs. This finding offers a new perspective on the development of digital health education, particularly the application of AI as a dynamic moderating factor, which facilitates the implementation of personalized and adaptive feedback loops, enhancing the precision and effectiveness of interventions. Although the study demonstrates the model’s efficacy, future research should further explore the personalized adjustment mechanisms of AI algorithms and their long-term effects, providing deeper theoretical support and practical guidance for health behavior change and quality of life improvement.

## 6 Limitations

Although this study employed a rigorous quasi-experimental randomized controlled trial, ensuring high internal validity, it still has certain limitations. First, the sample was sourced from a single hospital in one region, with participants sharing relatively similar health literacy, education levels, and acceptance of AI. This limits the generalizability of the findings across regions and cultures. Future studies should consider including diverse geographical locations and cultural backgrounds to assess the model’s universality. Second, the PBL-KAP-AI model has yet to be validated in other cancer types or chronic disease populations, and its applicability and broader use remain to be evaluated. Therefore, future research could expand to other disease groups to further explore its effectiveness.

Third, while the AI system used in this study relies on high-quality data and strict privacy protection measures, it does not yet possess adaptive intervention capabilities based on individual cognitive trajectories. Future research could integrate natural language processing and personalized modeling techniques to enhance the AI system’s ability to deliver precise and individualized interventions. Additionally, although the intervention duration was 3 months, it is difficult to assess the long-term maintenance of behavior changes and the subsequent impact on quality of life. Therefore, future research should conduct multi-timepoint longitudinal studies to track long-term effects and compare the acceptance and effectiveness of AI interventions across different cultural contexts, further verifying the sustainability and universality of the intervention.

## 7 Conclusion

This study constructs and validates an innovative spiral health education model that integrates the PBL teaching method, the KAP behavioral theory, and an AI-driven dynamic push mechanism. The model has been shown to significantly improve health behaviors, strengthen health beliefs, and enhance the quality of life in breast cancer patients. The study reveals a mediating mechanism through which educational satisfaction affects quality of life via health behaviors, and for the first time, demonstrates the moderating role of health beliefs. This model extends the conventional passive teaching approach by incorporating interactivity, adaptability, and sustainability, offering vast potential for future applications. As the model is extended to diverse populations, it is expected to promote the localization and systematic implementation of global health education, providing theoretical support and practical pathways for the development of digital health interventions. In summary, this study presents a novel theoretical framework and practical guidance for the innovative advancement of health education, emphasizing the critical role of health beliefs and behaviors in improving patients’ quality of life.

## Data Availability

The datasets presented in this article are not readily available because the datasets generated and analyzed during the current study are available from the corresponding author on reasonable request. All original contributions are included in the article and supplementary material. Requests to access the datasets should be directed to YL, 105359258@qq.com.
